# Career Adaptability, Work Engagement, and Employee Well-Being Among Chinese Employees: The Role of Guanxi

**DOI:** 10.3389/fpsyg.2019.01029

**Published:** 2019-05-14

**Authors:** Xuhua Yang, Yaqian Feng, Yuchen Meng, Yong Qiu

**Affiliations:** ^1^School of Labor Economics, Capital University of Economics and Business, Beijing, China; ^2^Business School, Beijing Technology and Business University, Beijing, China

**Keywords:** career adaptability, work engagement, employee well-being, guanxi, China

## Abstract

The present study examined whether and how career adaptability predicts employee well-being (EWB) based on career construction theory. A three-wave questionnaire design was used to collect the data, and 338 employees participated in the study. The results suggest that career adaptability has a significant effect on work engagement, which, in turn, predicts EWB. In addition to developing a mediation model, we tested the effect of guanxi as a moderator on the former part of the model. Thus, a moderated-mediation model was constructed in this research. In addition to the finding of the mediating role of work engagement, the discussion of guanxi represents a more important novel aspect that draws attention to contextual factors that may shape how employees respond to career adaptability. The results revealed that the indirect effect of career adaptability on EWB through work engagement when guanxi is low is stronger than that when guanxi is high. Furthermore, we discuss the limitations of this study and the implications for future research on career adaptability and EWB.

## Introduction

Currently, the career and job landscape is characterized by ongoing uncertainty and increasing instability and flexibility (Rudisill et al., 2010). Furthermore, pressure at work more broadly represents a growing threat to employee well-being (EWB) (Guest, 2017). Especially in China, as the workplace environment is becoming more market-oriented and competitive (Ren and Chadee, 2017), the increase in negative emotions among Chinese employees has emerged as a significant phenomenon (Zheng et al., 2015). Although concern regarding EWB is lacking in practice, it is important for organizations due to its association with positive organizational outcomes, such as improved performance and lower labor turnover (Daniel and Harris, 2000; Lyubomirsky et al., 2005; Proudfoot et al., 2009). Against this backdrop, EWB has recently been considered a beneficial supplement to human resource management (Guest, 2017). Thus, employees’ pursuit of well-being in the workplace is highly important (Ryan and Deci, 2001; Lent and Brown, 2008).

Scholars often use the concepts of psychological well-being (PWB) and subjective well-being (SWB) to represent employees’ overall well-being (Peng and Chen, 2010; Zheng et al., 2015). However, these concepts do not correctly represent EWB in China. In this research, we use a concept of EWB designed for Chinese employees. Prior research on the antecedents of well-being provides strong evidence regarding human resources policy and practice, which can be summarized by the development of human capacities, the provision of engaging work, positive social and physical environments, etc. (Guest, 2017). Hence, individuals are expected to build a repertoire of competencies as career adaptation tools (Ren et al., 2015), including regulation skills, adaptability, and self-awareness (Hall and Chandler, 2005; Savickas, 2005; Rudisill et al., 2010). In fact, researchers have carried out a series of studies on related issues necessary to meet the above needs. Career construction theory (CCT), which focuses on developing a repertoire of competencies and adaptively managing career paths (Savickas et al., 2009), is an innovative approach. The CCT framework (Savickas, 2013) accurately indicates that “adaptation” (positive outcomes in a changing situation) is fostered by “adaptability” (the ability to possess psychosocial resources for change) through the process of “adapting” (responding to various conditions). Actually, adaptation, or goodness of fit, is indicated by success, satisfaction, and development (Savickas and Porfeli, 2012). Previous studies have demonstrated that career adaptability has an effect on adapting and adaptation, such as career or job satisfaction (Chan and Mai, 2015; Coetzee and Stoltz, 2015; Fiori et al., 2015; Han and Rojewski, 2015), career or work engagement (Rossier et al., 2012; Nilforooshan and Salimi, 2016), career success (Zacher, 2014), and professional well-being (Maggiori et al., 2013). More specifically, psychological adaptation is conceptualized as “affective-emotional” and “cognitive-evaluative” well-being (Lent, 2004; Perera and McIlveen, 2014). Thus, by considering the psychological pressure caused by a changing workplace environment (Siu et al., 2005; Lu et al., 2011), we focus on well-being associated with environmental features as psychological adaptation outcomes and work engagement as an adapting process, by using the conceptual and integrative CCT framework in this paper.

In addition, adaptability is developed through the interaction between people’s inner and outer worlds. These worlds are closely related to specific roles and situations. Adaptability has been shown to have boundary conditions set by the culture environment. Prior research has primarily focused on the outcomes of career adaptability but overlooked important contextual factors. In the common workplace in China, guanxi suggests that interpersonal connections represent a stronger driver of typical relationships than rules and regulations in the Chinese culture (Zhang et al., 2015). This study explores indigenous guanxi culture in China, which is rooted in Confucianism, and describes personal and non-work-related connections that are reinforced implicitly by reciprocity and the exchange of favors (Chen et al., 2013). Given that guanxi is an influential philosophy deeply rooted in Chinese society, it is difficult to imagine the absence of guanxi in Chinese workplaces (Chen et al., 2013; Warner, 2013).

Therefore, this study contextualizes well-being in workplaces in which employees simultaneously manage work pressure and cultural-specific relationships. The aims of this study are as follows. First, we examine career adaptability as an antecedent of well-being based on CCT (Savickas, 1997, 2005). Career research in the Chinese context is at an embryonic stage (Russo et al., 2014; Ren and Chadee, 2017), and there is an urgent need to evaluate well-being in practice (Zheng et al., 2015). Second, we identify the mediation process through which career adaptability predicts employees’ well-being via work engagement. Third, we explore the boundary conditions by testing the moderation effect of guanxi based on situation strength theory (Mischel, 1977).

The study makes several contributions to the literature on career adaptability and well-being. First, this study focuses specifically on well-being to address the weaknesses of previous research. In China, academic research on EWB typically focuses on non-professionals (He and Pan, 2011; Xie, 2011; Yang, 2013) and still lags behind the needs of organizations (Zheng et al., 2015). In addition, previous research investigating well-being has failed to consider the integration of well-being, and the antecedents of well-being, i.e., individual characteristics and the environment, have not been considered in combination. However, changeable and flexible working environments emphasize the interaction between individuals and the environment and have resulted in the emergence of the career adaptability concept. Second, this study contributes to the small but growing body of literature on the importance of culture as a context for understanding career adaptability. This study considers the effects of the Chinese indigenous guanxi culture in the workplace by focusing specifically on guanxi as a boundary condition. Finally, a greater understanding of the antecedents, mechanisms, and boundary conditions of well-being allows for more accurate predictions of proactive human resource management in the context of culturally diverse workplaces. Although some studies have examined the impact of career adaptability on job satisfaction or general and professional well-being (Maggiori et al., 2013; Fiori et al., 2015), there is not enough focus on the adaptation of EWB and adapting to the mediating process, especially in China. Previous research has shown that career adaptability affects work engagement and well-being (Rossier et al., 2012; Maggiori et al., 2013); this paper integrates these concepts into a unified framework.

## Theoretical Background and Study Hypotheses

### Career Adaptability and EWB

The central goal of CCT is to obtain an understanding of how people use their work for self-improvement and to achieve satisfaction and happiness from social interaction (Savickas, 2002, 2005). This purpose is consistent with individual goals related to well-being (Walsh and Eggerth, 2005). Currently, work is a crucial part of most people’s lives that has a great influence on their personal well-being (Zheng et al., 2015). The working environment is very different from the general living environment. Therefore, the concept of EWB must be distinguished from general well-being. To date, researchers have not reached a consensus regarding the definition of EWB (Page and Vella-Brodrick, 2009). In general, SWB defines well-being as pleasure from the hedonic view (Diener, 1984, 2000; Diener and Ryan, 2009), while PWB stresses achievement from the eudemonic view (Ryff and Keyes, 1995; Ryff and Singer, 2008). EWB integrates SWB and PWB (Page and Vella-Brodrick, 2009; Peng and Chen, 2010) and extends the extant Western literature. In addition, in collectivist cultures, individuals are more willing to sacrifice their personal desires and focus on the group needs to improve the well-being of the group (Markus et al., 1996), which differs from the personal standards in Western countries (Diener, 1984, 2000). As a result, societal and others’ standards might play more important roles than personal standards in determining people’s well-being in Chinese societal and cultural circumstances. Thus, we use a concept of EWB designed for Chinese employees, which is defined as employees’ perceptions and feelings regarding their work, life, and psychological experience satisfaction exhibited in both their work and personal lives (Zheng et al., 2015).

Career construction theory regards adaptability as core psychosocial resources or transactional competencies necessary for achieving adaptation goals. This theory indicates that higher levels of adaptation are expected among those who are willing (adaptive) and able (adaptability) to perform behaviors in constantly changing work environments (Savickas and Porfeli, 2012). As an intervention model, CCT explains how individuals cultivate self-improvement and fulfillment through work (Savickas, 2002, 2005). Hence, provides a meaningful lens for understanding well-being, which is also involved in socially constructed outcome that individuals develop vis-à-vis their communities (Hartung and Taber, 2008).

Career adaptability is a psychosocial construct that denotes an individual’s resources for coping with current and anticipated tasks, transitions, and traumas in their occupational roles that alter their social integration to either a large or small degree (Savickas, 1997; Savickas and Porfeli, 2012). When individuals face a career choice or dilemma, career adaptability can help them enhance their sense of professional self-efficacy, eliminate decision-making difficulties (Urbanaviciute et al., 2014), achieve better performance (Ohme and Zacher, 2015), and obtain job and life satisfaction (Fiori et al., 2015). When employees fulfill their needs and achieve self-actualization, their gratification leads to happiness (Wilson, 1967). Thus, as a psychological adaptation, EWB could be affected by career adaptability. Furthermore, many previous studies have found that career adaptability is associated with life and professional satisfaction and SWB (Rossier et al., 2012; Maggiori et al., 2013; Rudolph et al., 2017; Ramos and Lopez, 2018). Thus, from the perspective of integrated well-being, we propose the following hypothesis:

**Hypothesis 1:** Career adaptability is positively related to EWB.

### The Mediating Role of Work Engagement

Adaptation is a consequence of adapting, which is defined as performing adaptive behaviors that address changing conditions (Ployhart and Bliese, 2006). Career adapting includes mastering vocational development tasks, coping with occupational transitions, and adjusting to work traumas and contingencies (Savickas and Porfeli, 2012; Hirschi and Valero, 2015). Thus, adaptive responses include associated “beliefs” or “behaviors” that individuals use to cope with job assignment and changing work conditions. According to the terminology of CCT, adaptation is fostered by adaptability resources via the process of adapting and adaptive responses. Thus, psychological adjustments are affected by adaptability through engagement coping.

Work engagement, which is defined as “the positive, fulfilling and work-related state of mind that is characterized by vigor, dedication, and absorption” (Van Wingerden et al., 2017), refers to a positive, affective-motivational state of high energy combined with high levels of dedication and a strong focus on work (Schaufeli et al., 2006). Work engagement has been shown to coincide with high levels of creativity, task performance, organizational citizenship behavior, financial results, and client satisfaction (Bakker and Albrecht, 2018). As a type of proactive adapting response (Merino-Tejedor et al., 2016), work engagement reflects proactive motivation (Hirschi et al., 2013). Work engagement is clearly one of the most important ways to achieve self-actualization through high effort, positive affect, and motivated cognition in the workplace, while well-being is regarded as the adapting result of self-fulfillment and self-improvement. Therefore, engagement has a positive effect on improving the mental well-being of employees (Matz-Costa et al., 2012; Robertson et al., 2012). In addition, the positive effect of work engagement on well-being has been supported by recent empirical research (Rossier et al., 2012; Salanova et al., 2014; Shimazu et al., 2015). Furthermore, career adaptability can be considered a social psychological resource that can be used to address career changes and leads to adaptive engaging responses (Nilforooshan and Salimi, 2016), which, in turn, lead to adaptation outcomes. Based on these findings, we argue that general adaptive resources could be positively related to work engagement, which, in turn, regulates individuals’ adapting response and eventually leads to positive well-being outcomes. Therefore, we propose the following hypothesis:

**Hypothesis 2:** Work engagement mediates the relationship between career adaptability and EWB.

### The Moderating Role of Guanxi

According to the CCT, the effect of career adaptability has boundary conditions (Savickas and Porfeli, 2012). Since this research focuses on the career process, we naturally pay attention to workplace relationships that are most typically observed in organizations. This study explicitly considers the sociocultural context to explain career adaptability and EWB by guanxi. Reflecting the flexibility and complexity of the Chinese language, the plethora of implicit and explicit definitions of guanxi challenges researchers. Literally, the Chinese term guanxi means “connections” or “relationships”. More concretely, guanxi refers to personal connections bound by implicit psychological contracts to exchange reciprocity, nurture mutual commitment, and aim for long-term relationships (Chen et al., 2013). Previous studies have used two basic approaches to define guanxi (Chen et al., 2004). The categorical approach views guanxi as a given when there are particular ties (Tsui and Farh, 1997). The dynamic approach views guanxi as the general quality of relationships (Wong et al., 2003). Because researchers suggest treating guanxi as a continuous variable, we remain consistent and describe guanxi as personal, non-work relationships (Chen et al., 2013) that can be built and used to progress through life and work (Xin and Pearce, 1996; Chen et al., 2013). We are interested in guanxi as informal, personal relationships rather than as formal, official relationships in organizations, such as kinships, birthplaces, alma maters, and work units, or as personal or demographic similarities (Tsui and Farh, 1997; Chen et al., 2004).

Traditional Western-based theories of supervisor-subordinate relationships tend to use the concept of leader-member exchange (LMX). This concept focuses on social exchanges in the work domain, while omitting potential exchanges that occur in the private domain. In contrast to LMX, guanxi in China mainly covers non-work exchanges within the vertical dyad, and the benefits of being involved in such exchanges can be social and economic in nature. The social exchanges embedded in guanxi are bound by personal favors, obligations, and trust (Farh et al., 1998; Ren and Chadee, 2016). Employees from societies undergoing substantial economic and social transitions and specifically those moving from the lack of bureaucracy at work toward increased bureaucracy, such as individuals in China, Russia, and Brazil, typically have high personal dependence on their supervisors beyond the work domain (Smith et al., 2012; Zhang et al., 2015). Past research indicates that guanxi is conceptually distinct from LMX (Law et al., 2000). The LMX perspective is particularly limited in explaining employee career development in the Chinese context (Ren and Chadee, 2016) in which relationships assume a broader meaning beyond work and are more appropriately captured by guanxi (Chen et al., 2014). CCT posits that employees need to interact with the environment during the process of adaptation and construction (Savickas, 2013). Thus, it is necessary to consider guanxi as an environmental boundary in career development because of the theoretical relevance of the cultural context and the blurred boundaries between work and non-work (Trefalt, 2013).

The level of guanxi represents the strength of the environment. Situation strength theory states that an individual’s perception of a situation could modulate the effect of their individual characteristics on their behavior or cognitive expression (Caspi and Moffitt, 1993; Meyer et al., 2010). Situations can be divided into strong situations and weak situations (Beaty et al., 2001; Cooper and Withey, 2009). A strong situation may limit the expression of personality (Cooper and Withey, 2009) because it can provide very clear guidelines regarding what constitutes valued work behaviors, which ultimately attenuate perception-performance validities. In contrast, a weak situation amplifies the effectiveness of the results because it can provide clues regarding expected behavior and, thus, can lead to behavioral expression that is consistent with basic personal tendencies, such as competency and abilities (McCrae and Costa, 1999). The CCT prefers to consider career adaptability as personality in terms of the socially constructed reputation that individuals develop vis-à-vis their communities. When employees are in a high guanxi context, the predictive validity of career adaptability is attenuated. Because a strong relationship with the leader motivates employees to engage in reciprocity and acknowledge the consciousness of return, the relationship also constrains employees’ behavior by an invisible bond. In contrast, when employees are in a low guanxi context, employees are not bound by an exchange relationship with their leaders; thus, the degree of their involvement in work depends more on their individual traits and personality. Therefore, the positive interaction effect between career adaptability and work engagement is enhanced.

Furthermore, previous research has shown that such a “relationship” refers to the beneficial rewards that employees receive from their superiors (Law et al., 2000), suggesting that employees could have a positive response as feedback. However, recently, some studies have found that employees’ perception of having a supportive boss is not necessarily an important predictor of positive behavior and may even be a negative predictor (Burnett et al., 2015). Previous studies have shown that supervisor support is not related to higher personal initiative (Frese and Fay, 2001). A recent study conducted by Ren and Chadee (2017) further supported a similar conclusion and showed that the effect of guanxi on employee self-development diminished after reaching an inflection point based on the Chinese context, suggesting that too much guanxi is not necessarily good for employees and may even have a negative influence under constraint conditions. A high level of guanxi could create excessive pressures on employees because of implied reciprocity beyond their capacity. In summary, the effects of personality on motivational processes were more pronounced when the subjects had the freedom to express themselves (Hollenbeck et al., 1989) without much situational influence. We accordingly propose the following hypotheses:

**Hypothesis 3:** Guanxi negatively moderates the relationship between career adaptability and work engagement, and thus, the positive effect is stronger when guanxi is at a low level.

**Hypothesis 4:** The indirect effect of career adaptability on EWB via work engagement is moderated by guanxi, and thus, the indirect effect is stronger when guanxi is at a low level.

**FIGURE 1:**
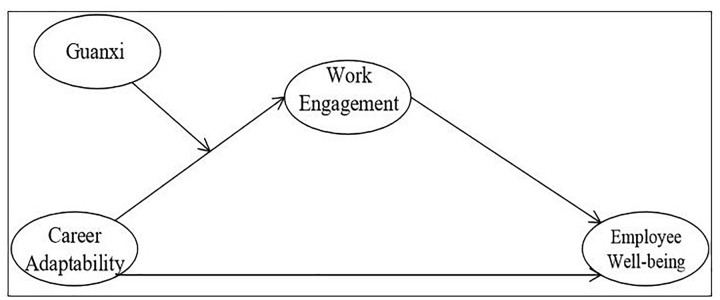
The proposed moderated-mediation model.

Thus, we examined moderated-mediation models of the relationship among these variables as shown in Figure 1.

## Materials and Methods

### Procedures and Participants

This research was conducted through a survey using anonymous questionnaires. The eligible participants were full-time employees working in China at four state-owned enterprises to ensure the representativeness of the samples. After obtaining approval from the four companies, we mailed the questionnaires and e-mailed the guidelines to each company. In the guidelines, we informed the participants that their responses would remain anonymous. Based on assistance from the human resource management department of each enterprise, the questionnaires were distributed to individuals who voluntarily agreed to participate. In addition, data were collected from the employees at three time points over 2 months to minimize potential common-method biases (Podsakoff et al., 2012). This study was carried out in accordance with the Declaration of Helsinki and ethical guidelines and approved by the Human Research Ethics Committee (HREC) at the School of Labor Economics, Capital University of Economics and Business. Written informed consent was obtained from all participants.

A return envelope with seal tape was provided to each participant for the finished questionnaire to maintain confidentiality. Initially, 452 participants completed the first survey concerning their career adaptability and demographic information; 411 responses were retained after removing 41 responses due to low efforts in the responses (i.e., 80% of the responses were the same answers or quick answers were given). After 4 weeks, we conducted the second wave of the survey. Internal media were used again to connect with the employees that participated during the first wave of the survey and collect information regarding work engagement and guanxi. Overall, 379 employees completed the second survey. The final wave was conducted after 1 month, and we collected information regarding EWB. Finally, after removing all incomplete, mismatched, and missing cases, 338 matched responses were available from all time points for the analyzes. In this final sample, the participants were 38.28 years old (*SD* = 9.66), had at least a bachelor’s degree (53.8%), had 7.75 years (*SD* = 7.84) of organizational tenure, and had 4.98 years (*SD* = 5.77) of position tenure. Most participants were male (72.2%) because the participants were mostly employed at heavy industrial companies that had more male employees. The analyzes were performed using the Statistical Package for Social Sciences version 24.0 (IBM SPSS Statistics 24, SPSS Inc., Chicago, IL, United States) and the AMOS statistical package version 22.0 (Arbuckle, 2010).

### Measures

According to Brislin’s (1970) back-translation procedure, the original survey items were translated into Chinese and then back-translated into English. In this study, all survey items were assessed on a five-point Likert-type scale ranging from strongly disagree to strongly agree.

#### Career Adaptability

The Career Adapt-Abilities Scale-Short Form (CAAS-SF) consisting of 12 items developed and validated by Maggiori et al. (2017) was used to assess career adaptability. This measure is a simplified version of the original questionnaire that included 24 items (Savickas and Porfeli, 2012). The short questionnaire has been used to assess career adaptability in different country contexts, as the original. The scale of the CAAS-SF in the French/German version had a reliability of 0.90/0.90 (Maggiori et al., 2017). For convenience, the CAAS-SF was used in this study. The overall scale in China in this sample had a reliability of 0.93, which is higher than that in the international sample. One sample item is “Becoming aware of the educational and vocational choices that I must make.”

#### Work Engagement

The short form of the Utrecht Work Engagement Scale (UWES-9) developed and validated by Schaufeli et al. (2006) was used to assess work engagement. This instrument was developed to measure vigor, dedication, and absorption, with three items each. Previous studies have reported that the Cronbach’s α of the scale ranged between 0.85 and 0.92 across samples from 10 countries (Schaufeli et al., 2002). The Cronbach’s α reliability estimate of the scale in the present study was 0.92. One sample item is “At my work, I feel bursting with energy.”

#### Employee Well-Being

The EWB scale developed and validated by Zheng et al. (2015) was used. Eighteen items were used to evaluate the individuals’ overall satisfaction with their life well-being, workplace well-being, and PWB overall. Sample items include “I feel satisfied with my life,” “Work is a meaningful experience for me,” and “I generally feel good about myself, and I’m confident”. Regarding the internal reliability, data from two studies indicate values of 0.93 and 0.94, respectively (Zheng et al., 2015). The internal consistency in the present study was 0.94, which is comparable to previous findings.

#### Guanxi

Guanxi was assessed by a four-item scale developed by Chen et al. (2009). The scale is used to analyze the specific, private relationship between a supervisor and subordinate. The alpha coefficient of guanxi in the present study was 0.87, which is comparable to previous findings ranging from 0.79 to 0.87 (Chen et al., 2009, 2012). One sample item is “My supervisor would ask me to help him/her deal with some family errands.”

#### Control Variables

Because demographic backgrounds may have potential confounding effects (Becker, 2005), we used the following demographic variables as control variables in our model: age, gender, education level, and seniority in organizations and jobs.

## Results

### Preliminary Analyzes

We began by conducting a confirmatory factor analysis (CFA) with AMOS 22.0 to estimate the model. In the CFA analysis, the goodness of fit of our model was evaluated with a variety of fit metrics as follows: χ2/df = 2.549, *p* < 0.001; comparative fit index (CFI) = 0.901; incremental fit index (IFI) = 0.901; root mean square error of approximation (RMSEA) = 0.068; and root mean square residual (RMR) = 0.047. The results of the CFA showed that the proposed model fit the data as the normalized chi-square (chi-square/degrees of freedom) of the CFA model was lower than the recommended value of 3.0, and the values of the CFI and IFI were higher than 0.90. The RMSEA was lower than 0.08, and the RMR was lower than 0.05.

The convergent validity of a model can be validated by the criteria that all average variance extracted (AVE) exceed 0.50 (Fornell and Larcker, 1981). Furthermore, if the component reliability (CR) is at least 0.70, convergent validity could be present. According to the analytical results of the AVE and CR, all AVEs of the research variables were higher than 0.60 as follows: 0.80 (career adaptability), 0.89 (work engagement), 0.80 (EWB), and 0.68 (guanxi). Additionally, all CRs exceeded 0.80 as follows: 0.94 (career adaptability), 0.96 (work engagement), 0.92 (EWB), and 0.89 (guanxi). These results reveal that the convergent validity of the research variables in this study is satisfactory.

### Descriptive Analysis

Table 1 displays the means, standard deviations, and correlation coefficients of the variables. Consistent with the above hypotheses, career adaptability was positively correlated with EWB (*r* = 0.483, *p* < 0.01) and work engagement (*r* = 0.575, *p* < 0.001), which was positively correlated with EWB (*r* = 0.671, *p* < 0.001). Guanxi was significantly and positively related to work engagement (*r* = 0.576, *p* < 0.001) and EWB (*r* = 0.532, *p* < 0.001). In addition, several control variables were positively related to the main research variables, including a correlation between gender and guanxi (*r* = 0.109, *p* < 0.05) and a correlation between gender and career adaptability (*r* = 0.207, *p* < 0.01).

**Table 1:** Means, standard deviations, and correlations.

Variables	Mean	*SD*	1	2	3	4	5	6	7	8
(1) Gender^a^	0.28	0.45								
(2) Education^b^	4.48	0.78	0.115^*^							
(3) Age	38.28	9.66	-0.494^**^	-0.025						
(4) Organization tenure (years)	7.75	7.84	-0.287^**^	-0.002	0.405^**^					
(5) Position tenure (years)	4.98	5.77	-0.253^**^	-0.003	0.301^**^	0.480^**^				
(6) Career adaptability	3.91	0.66	0.207^**^	-0.054	-0.156^**^	-0.128^*^	0.012			
(7) Work engagement	3.68	0.79	0.106	-0.015	-0.018	-0.161^**^	-0.053	0.575^**^		
(8) Guanxi	3.35	0.88	0.109^*^	-0.217^*^	-0.077	-0.080	-0.046	0.381^**^	0.576^**^	
(9) Employee well-being	3.73	0.69	0.102	-0.066	0.008	-0.118^*^	-0.177^**^	0.483^**^	0.671^**^	0.532^**^


*n = 338; ^*a*^0 = male, 1 = female; ^*b*^1 = high school or below, 2 = associate, 3 = bachelor’s, 4 = master’s or above; ^∗^*p* < 0.05. ^∗∗^*p* < 0.01.*

### Hypothesis Testing

The SPSS-PROCESS was used to test the hypotheses as proposed by Hayes (2013) and tested by Cole et al. (2008). PROCESS offers many of the features of SOBEL, INDIRECT, MODPROBE, and MODMED and includes the complexity of models combining moderation and mediation. PROCESS can be used to facilitate estimations of indirect effects by using the SOBEL test (Sobel, 1982) and a bootstrap approach (Preacher and Hayes, 2004) to obtain the confidence interval (CI) and incorporate the stepwise procedure suggested by Baron and Kenny (1986). To test these effects, Models 4 and 7 in the PROCESS bootstrapping approach provided by Hayes were chosen to test the model as proposed in previous studies (Hirschi et al., 2013).

#### Step 1

Using the procedure proposed by Preacher and Hayes (2008), we tested the direct effect of career adaptability on EWB and the indirect effect of career adaptability on EWB via work engagement. First, we examined the relationship between the independent and mediation variables. After controlling for the effects of gender, age, education, organizational tenure, and job tenure, the results showed that career adaptability (*B* = 0.69, *t* = 12.70, *p* < 0.001) was a significant direct predictor of work engagement. Second, after controlling for the effects of career adaptability, work engagement had a significant effect on EWB (*B* = 0.50, *t* = 11.71, *p* < 0.001), and career adaptability had a reduced relationship with EWB (*B* = 0.19, *t* = 3.60, *p* < 0.001). Therefore, work engagement partially mediated career adaptability and EWB. Furthermore, by using the bootstrapping method for further calculation, we found that career adaptability had a significant indirect effect of 0.34 (95% CI = [0.27,0.44]) and a significant direct effect of 0.19 (95% CI = [0.08,0.29]) on EWB through the mediation of work engagement; these results did not include 0. These results support Hypotheses 1 and 2 (see Tables 2A,B).

**Table 2A:** Mediation effects of work engagement on the relationship between career adaptability and employee well-being.

Variable	Work engagement as dependent variable	Employee well-being as dependent variable	
		
	Model 2	Model 1	Model 3
Constant	0.639	1.254	0.935
Gender	0.032	0.035	0.019
Education	0.007	0.016	0.013
Age	0.011	0.012	0.006
Organization tenure (years)	-0.012	-0.001	0.005
Position tenure (years)	-0.006	-0.027	-0.024
Career adaptability	0.689^***^	0.530^***^	0.186^***^
Work engagement			0.500^***^
*F*	30.246^***^	22.361^***^	46.783^***^
*R*^2^	0.354	0.289	0.498


*n = 338. ^∗^*p* < 0.05, ^∗∗^*p* < 0.01, and ^∗∗∗^*p* < 0.001.*

**Table 2B:** Mediation effects of work engagement in the relationship between career adaptability and employee well-being.

Direct and indirect effect of career adaptability on employee well-being				
	**Effect**	**Boot SE**	**Boot LLCI**	**Boot ULCI**

Direct effect	0.186	0.051	0.084	0.287
Indirect effect	0.344	0.044	0.268	0.442


*n = 338.*

#### Step 2

To examine the moderation and moderated-mediation models, we used the procedure developed by Preacher et al. (2007). In this two-equation procedure, one equation is used for the “mediator model” (work engagement as a dependent variable), and the other equation is used for the “dependent variable model” (EWB as a dependent variable). To support the simple moderation hypothesis (H3), the coefficients of the interaction terms in the mediator models should be significant. To support the moderated-mediation models, the indirect effects should vary with different levels of the moderators (H4). After controlling for the effects of gender, age, education, organizational tenure, and job tenure, the results showed that the effect of the interaction between career adaptability and guanxi on work engagement (*B* = -0.16, SE = 0.04, *t* = -3.85, *p* < 0.001) was significant, supporting H3 (see Table 3A).

**Table 3A:** Moderation and moderated mediation effects of guanxi.

Mediator variable model with work engagement as the dependent variable						
**Variable**	***B***	**SE**	***t***	***P***	**LLCI**	**ULCI**

Constant	3.487	0.292	11.925	0.000	2.912	4.062
Career adaptability	0.493	0.050	9.912	0.000	0.395	0.591
Guanxi	0.400	0.038	10.654	0.000	0.326	0.474
CA × guanxi	-0.162	0.042	-3.854	0.000	-0.245	-0.079
Gender	0.169	0.069	2.458	0.015	0.034	0.305
Education	-0.023	0.045	-0.516	0.606	-0.112	0.066
Age	0.011	0.004	2.894	0.004	0.004	0.018
Organization tenure (years)	-0.013	0.005	-2.841	0.005	-0.022	-0.004
Position tenure (years)	-0.005	0.006	-0.886	0.376	-0.017	0.007
*R*	0.729					
*R*^2^	0.532					


*n = 338.*

**FIGURE 2:**
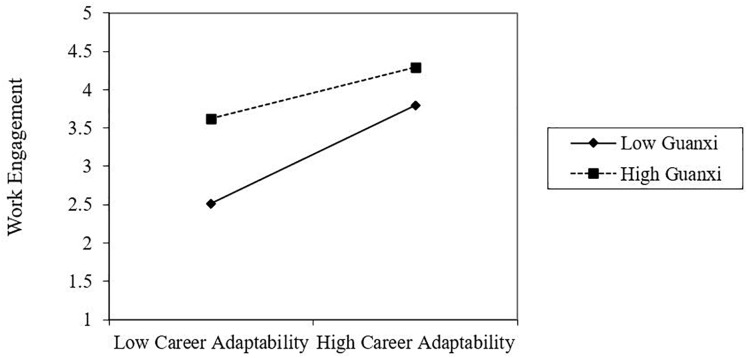
Effect of the interaction between career adaptability and guanxi on work engagement. Note: Low guanxi is defined as at least one standard deviation below the mean; high guanxi is defined as at least one standard deviation above the mean. Low numbers indicate greater work engagement.

To clearly illustrate the interaction effects, the interaction was plotted at one standard deviation below and above the mean of guanxi. As shown in Figure 2, when guanxi was low, the relationship between career adaptability and work engagement was significant, *B* = 0.64, SE = 0.06, *t* = 10.48, *p* < 0.001. When guanxi was high, the effect was diminished, *B* = 0.35, SE = 0.06, *t* = 5.54, *p* < 0.001.

Further tests of the moderated-mediation effects of guanxi using the PROCESS bootstrapping approach proposed by Hayes (2013) showed that the indirect effect (CI = [-0.15, -0.03]) was significant. Furthermore, the results showed that the indirect effect of career adaptability on EWB through work engagement significantly varies between the high and low levels of guanxi, suggesting that the indirect effect of career adaptability on EWB was smaller at a high level of guanxi than the indirect effect at a low level of guanxi (see Table 3B), supporting H4. Thus, we conclude that the moderated-mediation effect was supported.

**Table 3B:** Moderation and moderated mediation effects of guanxi.

Conditional indirect effect as a function of guanxi				
**Value of guanxi**	**Indirect effect**	**Boot SE**	**Boot LLCI**	**Boot ULCI**

Mean - 1 SD(-0.88)	0.318	0.048	0.237	0.430
Mean(0)	0.247	0.034	0.186	0.318
Mean + 1 SD(0.88)	0.175	0.039	0.110	0.265

**Index of moderated mediation**				

**Mediator**	**Index**	**Boot SE**	**Boot LLCI**	**Boot ULCI**

Work engagement	-0.081	0.032	-0.152	-0.028


*n = 338.*

## Discussion

### Main Findings

Using a sample of Chinese employees, this research tested whether and how career adaptability predicts work engagement and EWB. This research highlighted three main points. First, career adaptability plays a significant role in predicting work engagement and EWB. Second, work engagement plays a mediating role between career adaptability and EWB. Finally, guanxi plays a moderating role in this model. Specifically, the mediation model of career adaptability on EWB through work engagement was supported when employees experienced a low level of guanxi.

### Theoretical Implications

The results of the current study are consistent with previous research, and this study makes several contributions to the literature. First, based on CCT, we found that career adaptability is positively related to engagement adapting and well-being adaptation. In addition, the findings supplement previous studies (e.g., Rossier et al., 2012; Rudolph et al., 2017) showing the positive impact of adaptability on work outcomes and personal well-being. Career adaptability resources should be viewed as self-regulatory, psychosocial competencies that shape adaptive strategies and actions aiming to achieve adaptation goals in a volatile and complex environment (Savickas and Porfeli, 2012). This paper answered the call for a better understanding of how and why career adaptability leads to beneficial outcomes (Johnston, 2016). Employees who become more adaptable easily immerse themselves and focus on work; thus, it is easier for these employees to have positive experiences and positive feelings and pursue personal well-being. Furthermore, the mediation of work engagement has attracted researchers’ interests to the psychological mechanism underlying the career adaptability and EWB association.

Second, the findings were interpreted according to Chinese social and cultural values, which supplemented the boundary condition of career adaptability research. Because of the guanxi orientation in Chinese values, Chinese employees particularly value the gain and loss of relationships (Hwang, 1987). In contrast to previous studies, our results suggest that guanxi weakens the effect of career adaptability on work engagement and its indirect effect on EWB via work engagement. This study responds to the call of scholars to explore the context and boundary of career adaptability. In addition, adaptability resources can be viewed as human capital, which is defined as the accumulated competencies and knowledge gained through education and experience (Savickas and Porfeli, 2012), which are the special attributes of each individual. In general, employees do not experience more engagement or satisfaction based on personal adaptability when they perceive high guanxi. In contrast, adaptive employees exhibited an enhance engaging response when they perceive low guanxi as they think that is it still needed to master new skills, complete challenging tasks, and take greater responsibility to reach self-actualization. To be valued in an organization and competitive on the labor market, more adaptive employees will choose to increase their personal efforts to achieve greater well-being.

### Practical Implications

We can draw some practical implications from our research results. The first set of practices concerns investment in employees. As career adaptability is gaining increasing attention in the workplace, it is necessary for supervisors to provide multiple career paths to employees, especially in China. Enhancing competence through training and providing a sense of an attractive career future contribute to the feelings of security and well-being (Guest, 2017). To achieve this goal, leaders should design specific career plans, such as job enlargement and job rotation in the organization, and focus on self-planning, self-awareness, curiosity, and self-confidence.

The second set of practices concerns the provision of engaging work. Previous analyzes of the antecedents of well-being highlighted the importance of opportunities for control, skill use, and variety at work. Grote and Guest (2017) emphasized the need to accommodate and tap into individual proactivity. Moreover, some studies demonstrate the effectiveness of positive psychology interventions in proactively adapting to work (Costantini and Sartori, 2018). Thus, by providing employees the opportunity to participate in positive interventions aiming to improve their cognitive resources, employees can acquire a higher perception of positive emotions and states of work and increased awareness of the opportunities to adjust their characteristics in the workplace. Thus, organizations can promote positive work attitudes in employees, which can contribute to work engagement and well-being at work.

The third set of HR practices focuses on the creation of a positive social and physical environment. The moderating role of guanxi indicates that caution should be taken in cases in which employees exert effort to become obsequious instead of focusing on their work. Managers should address supervisor-subordinate guanxi to balance organizational incentives and employee expectations regarding employment. In cases in which adaptive employees reduce their work engagement, employers should adjust the resource availability according to their foreseeable personal contribution.

### Limitations and Future Research Directions

This study has several limitations. First, although we investigated the variables at intervals, career adaptability, and EWB are not static, and more stringent measures should be used to consider their dynamic relationship. In future research, longitudinal designs or experiments should be designed to better establish causality.

Second, we hope that researchers from other cultures can replicate our research. Then, we could consider multiple cultural factors in interpreting our results in the future. Our sample from China limits the universality of our conclusions. We believe that the relationship between superiors and subordinates in the workplace may help explain whether the pursuit of well-being interferes with the effect of guanxi.

Third, control over the baseline measures of the mediator and dependent variable is lacking because of the limitations of the investigation. In future research, we aim to further improve our research design and supplement the control variables to explore the real influence of the relationship in the model. In addition, future studies could continue to focus on the influence of gender and other control variables on the research model.

## Ethics Statement

All procedures performed in studies involving human participants were performed in accordance with the ethical standards of the institutional and/or national research committee and the 1964 Helsinki Declaration and its later amendments or comparable ethical standards with written informed consent from all subjects. This research was approved by the Human Research Ethics Committee at School of Labor Economics, Capital University of Economics, and Business.

## Author Contributions

XY and YQ designed the research and collected the data for the study. YF and YM analyzed the data and drafted the manuscript. All authors critically reviewed and approved the final version of this manuscript.

## Conflict of Interest Statement

The authors declare that the research was conducted in the absence of any commercial or financial relationships that could be construed as a potential conflict of interest.

## References

[B1] Arbuckle J. L. (2010). *IBM SPSS Amos 19 User’s Guide.* Crawfordville, FL: Amos Development Corporation, 635.

[B2] Bakker A. B. Albrecht S. (2018). Work engagement: current trends. *Career Dev. Int.* 23 4–11. 10.1108/CDI-11-2017-0207

[B3] Baron R. M. Kenny D. A. (1986). The moderator–mediator variable distinction in social psychological research: conceptual, strategic, and statistical considerations. *J. Pers. Soc. Psychol.* 51:1173 10.1037/0022-3514.51.6.1173 3806354

[B4] Beaty J. C. Jr. Cleveland J. N. Murphy K. R. (2001). The relation between personality and contextual performance in” strong” versus” weak” situations. *Hum. Perform.* 14 125–148. 10.1207/S15327043HUP1402_01

[B5] Becker T. E. (2005). Potential problems in the statistical control of variables in organizational research: a qualitative analysis with recommendations. *Organ. Res. Methods* 8 274–289. 10.1177/1094428105278021

[B6] Brislin R. W. (1970). Back-translation for cross-cultural research. *J. Cross Cult. Psychol.* 1 185–216. 10.1177/135910457000100301

[B7] Burnett M. F. Chiaburu D. S. Shapiro D. L. Li N. (2015). Revisiting how and when perceived organizational support enhances taking charge: an inverted U-shaped perspective. *J. Manag.* 41 1805–1826. 10.1177/0149206313493324

[B8] Caspi A. Moffitt T. E. (1993). When do individual differences matter? A paradoxical theory of personality coherence. *Psychol. Inquiry* 4 247–271. 10.1207/s15327965pli0404_1

[B9] Chan S. H. J. Mai X. (2015). The relation of career adaptability to satisfaction and turnover intentions. *J. Vocat. Behav.* 89 130–139. 10.1016/j.jvb.2015.05.005

[B10] Chen C. C. Chen X. P. Huang S. (2013). Chinese guanxi: an integrative review and new directions for future research. *Manag. Organ. Rev.* 9 167–207. 10.1111/more.12010

[B11] Chen C. C. Chen Y. R. Xin K. (2004). Guanxi practices and trust in management: a procedural justice perspective. *Organ. Sci.* 15 200–209. 10.1287/orsc.1030.0047

[B12] Chen Y. Friedman R. Yu E. Fang W. Lu X. (2009). Supervisor–subordinate guanxi: developing a three-dimensional model and scale. *Manag. Organ. Rev.* 5 375–399. 10.1111/j.1740-8784.2009.00153.x

[B13] Chen Y. Friedman R. Yu E. Sun F. (2012). Examining the positive and negative effects of guanxi practices. *SSRN Electron. J.* 28 715–735. 10.2139/ssrn.2071329

[B14] Chen Y. Yu E. Son J. (2014). Beyond leader–member exchange (LMX) differentiation: an indigenous approach to leader–member relationship differentiation. *Leadersh. Q.* 25 611–627. 10.1016/j.leaqua.2013.12.004

[B15] Coetzee M. Stoltz E. (2015). Employees’ satisfaction with retention factors: exploring the role of career adaptability. *J. Vocat. Behav.* 89 83–91. 10.1016/j.jvb.2015.04.012

[B16] Cole M. S. Walter F. Bruch H. (2008). Affective mechanisms linking dysfunctional behavior to performance in work teams: a moderated mediation study. *J. Appl. Psychol.* 93:945. 10.1037/0021-9010.93.5.945 18808218

[B17] Cooper W. H. Withey M. J. (2009). The strong situation hypothesis. *Pers. Soc. Psychol. Rev.* 13 62–72. 10.1177/1088868308329378 19144905

[B18] Costantini A. Sartori R. (2018). The intertwined relationship between job crafting, work-related positive emotions, and work engagement. Evidence from a positive psychology intervention study. *Open Psychol. J.* 11 210–221. 10.2174/1874350101811010210

[B19] Daniel K. Harris C. (2000). ‘Work, psychological well-being and performance’. *Occup. Med.* 50 304–309. 10.1093/occmed/50.5.304 10975125

[B20] Diener E. (1984). Subjective well-being. *Psychol. Bull.* 95:542 10.1037/0033-2909.95.3.542 6399758

[B21] Diener E. (2000). Subjective well-being: the science of happiness and a proposal for a national index. *Am. Psychol.* 55:34. 10.1037//0003-066X.55.1.34 11392863

[B22] Diener E. Ryan K. (2009). Subjective well-being: a general overview. *South Afr. J. Psychol.* 39 391–406. 10.1177/008124630903900402

[B23] Farh J.-L. Tsui A. S. Xin K. Cheng B.-S. (1998). The influence of relational demography and guanxi: the Chinese case. *Organ. Sci.* 9 471–488. 10.2307/2640274

[B24] Fiori M. Bollmann G. Rossier J. (2015). Exploring the path through which career adaptability increases job satisfaction and lowers job stress: the role of affect. *J. Vocat. Behav.* 91 113–121. 10.1016/j.jvb.2015.08.010

[B25] Fornell C. Larcker D. F. (1981). Evaluating structural equations models with unobservable variables and measurement error. *J. Mark. Res.* 18 39–50. 10.2307/3151312

[B26] Frese M. Fay D. (2001). Personal initiative: an active performance concept for work in the 21st century. *Res. Organ. Behav.* 23 133–187. 10.1016/S0191-3085(01)23005-6

[B27] Grote G. Guest D. (2017). The case for reinvigorating quality of working life research. *Hum. Relat.* 70 149–167. 10.1177/0018726716654746

[B28] Guest D. E. (2017). Human resource management and employee well-being: towards a new analytic framework. *Hum. Resour. Manag. J.* 27 22–38. 10.1111/1748-8583.12139

[B29] Hall D. T. Chandler D. E. (2005). Psychological success:When the career is a calling. *J. Organ. Behav.* 26 155–176. 10.1002/job.301

[B30] Han H. Rojewski J. W. (2015). Gender-specific models of work-bound Korean adolescents’ social supports and career adaptability on subsequent job satisfaction. *J. Career Dev.* 42 149–164. 10.1177/0894845314545786

[B31] Hartung P. J. Taber B. J. (2008). Career construction and subjective well-being. *J. Career Assess.* 16 75–85. 10.1177/1069072707305772

[B32] Hayes A. F. (2013). *Mediation, Moderation, and Conditional Process Analysis. Introduction to Mediation, Moderation, and Conditional Process Analysis: A Regression-Based Approach edn.* New York, NY: Guilford Publications, 1–20.

[B33] He L. X. Pan C. Y. (2011). Uncover the “Easterlin Paradox” of China: income gap, inequality of opportunity and happiness. *Manag. World* 8 11–22. 10.19744/j.cnki.11-1235/f.2011.08.003

[B34] Hirschi A. Lee B. Porfeli E. J. Vondracek F. W. (2013). Proactive motivation and engagement in career behaviors: investigating direct, mediated, and moderated effects. *J. Vocat. Behav.* 83 31–40. 10.1016/j.jvb.2013.02.003

[B35] Hirschi A. Valero D. (2015). Career adaptability profiles and their relationship to adaptivity and adapting. *J. Vocat. Behav.* 88 220–229. 10.1016/j.jvb.2015.03.010

[B36] Hollenbeck J. R. Williams C. R. Klein H. J. (1989). An empirical examination of the antecedents of commitment to difficult goals. *J. Appl. Psychol.* 74 18–23. 10.1037/0021-9010.74.1.18

[B37] Hwang K. K. (1987). Face and favor: the Chinese power game. *Am. J. Sociol.* 92 944–974. 10.1086/228588

[B38] Johnston C. S. (2016). A systematic review of the career adaptability literature and future outlook. *J. Career Assess.* 26 3–30. 10.1177/1069072716679921

[B39] Law K. S. Wong C. S. Wang D. X. Wang L. H. (2000). Effect of supervisor–subordinate guanxi on supervisory decisions in China: an empirical investigation. *Int. J. Hum. Resour. Manag.* 11 751–765. 10.1080/09585190050075105

[B40] Lent R. W. (2004). Toward a unifying theoretical and practical perspective on well-being and psychosocial adjustment. *J. Counsel. Psychol.* 51 482–509. 10.1037/0022-0167.51.4.482

[B41] Lent R. W. Brown S. D. (2008). Social cognitive career theory and subjective well-being in the context of work. *J. Career Assess.* 16 6–21. 10.1177/1069072707305769

[B42] Lu L. Kao S. F. Siu O. L. Lu C. Q. (2011). Work stress, Chinese work values, and work well-being in the greater China. *J. Soc. Psychol.* 151 767–783. 10.1080/00224545.2010.538760 22208113

[B43] Lyubomirsky S. King L. Diener E. (2005). ‘The benefits of frequent positive affect: does happiness lead to success?’ *Psychol. Bull.* 131 803–855. 10.1037/0033-2909.131.6.803 16351326

[B44] Maggiori C. Johnston C. S. Krings F. Massoudi K. Rossier J. (2013). The role of career adaptability and work conditions on general and professional well-being. *J. Vocat. Behav.* 83 437–449. 10.1016/j.jvb.2013.07.001

[B45] Maggiori C. Rossier J. Savickas M. L. (2017). Career adapt-abilities scale–short form (CAAS-SF) construction and validation. *J. Career Assess.* 25 312–325. 10.1177/1069072714565856

[B46] Markus H. R. Kitayama S. Heiman R. T. (1996). *Culture and “Basic” Psychological Principles.* New York, NY: Guilford.

[B47] Matz-Costa C. Besen E. Boone James J. Pitt-Catsouphes M. (2012). Differential impact of multiple levels of productive activity engagement on psychological well-being in middle and later life. *Gerontologist* 54 277–289. 10.1093/geront/gns148 23213083

[B48] McCrae R. R. Costa P. T. Jr. (1999). A five-factor theory of personality. *Handb. Pers. Theor. Res.* 2 139–153. 10.1007/978-1-4615-0763-5_11

[B49] Merino-Tejedor E. Hontangas P. M. Boada-Grau J. (2016). Career adaptability and its relation to self-regulation, career construction, and academic engagement among Spanish university students. *J. Vocat. Behav.* 93 92–102. 10.1016/j.jvb.2016.01.005

[B50] Meyer R. D. Dalal R. S. Hermida R. (2010). A review and synthesis of situational strength in the organizational sciences. *J. Manag.* 36 121–140. 10.1177/0149206309349309

[B51] Mischel W. (1977). “The interaction of person and situation,” in *Personality at the Crossroads. Current Issues in Interactional Psychology*, eds Endler N. S. Magnusson D. (New Jersey, NJ: Lawrence Erlbaum Associates), 333–352.

[B52] Nilforooshan P. Salimi S. (2016). Career adaptability as a mediator between personality and career engagement. *J. Vocat. Behav.* 94 1–10. 10.1016/j.jvb.2016.02.010

[B53] Ohme M. Zacher H. (2015). Job performance ratings: the relative importance of mental ability, conscientiousness, and career adaptability. *J. Vocat. Behav.* 87 161–170. 10.1016/j.jvb.2015.01.003

[B54] Page K. M. Vella-Brodrick D. A. (2009). The “what,” “why” and “how” of employee well-being: a new model. *Soc. Indic. Res.* 90 441–458. 10.1007/s11205-008-9270-3

[B55] Peng Y. Chen H. (2010). Reconstruction on the connotation of well-being based on an integrative perspective. *Adv. Psychol. Sci.* 18 1052–1061.

[B56] Perera H. N. McIlveen P. (2014). The role of optimism and engagement coping in college adaptation: a career construction model. *J. Vocat. Behav.* 84 395–404. 10.1016/j.jvb.2014.03.002

[B57] Ployhart R. E. Bliese P. D. (2006). “Individual adaptability (I-Adapt) theory: conceptualizing the antecedents, consequences, and measurement of individual differences in adaptability,” in *Understanding Adaptability: A Prerequisite for Effective Performance within Complex Environments* Vol. 6 eds Burke S. C. Pierce L. G. (Bingley: Emerald Group Publishing Limited).

[B58] Podsakoff P. M. MacKenzie S. B. Podsakoff N. P. (2012). Sources of method bias in social science research and recommendations on how to control it. *Annu. Rev. Psychol.* 63 539–569. 10.1146/annurev-psych-120710-100452 21838546

[B59] Preacher K. J. Hayes A. F. (2004). SPSS and SAS procedures for estimating indirect effects in simple mediation models. *Behav. Res. Methods Instru. Comput.* 36 717–731. 10.3758/bf03206553 15641418

[B60] Preacher K. J. Hayes A. F. (2008). Asymptotic and resampling strategies for assessing and comparing indirect effects in multiple mediator models. *Behav. Res. Methods* 40 879–891. 10.3758/BRM.40.3.87 18697684

[B61] Preacher K. J. Rucker D. D. Hayes A. F. (2007). Addressing moderated mediation hypotheses: theory, methods, and prescriptions. *Multivariate Behav. Res.* 42 185–227. 10.1080/00273170701341316 26821081

[B62] Proudfoot J. Corr P. Guest D. Dunn P. (2009). Cognitive-behavioural training to change attributional style improves employee well-being, job satisfaction, productivity and turnover. *Pers. Individ. Differ.* 46 147–153. 10.1016/j.paid.2008.09.018

[B63] Ramos K. Lopez F. G. (2018). Attachment security and career adaptability as predictors of subjective well-being among career transitioners. *J. Vocat. Behav.* 104 72–85. 10.1016/j.jvb.2017.10.004

[B64] Ren S. Chadee D. (2016). Influence of work pressure on proactive skill development in China: the role of career networking behavior and Guanxi HRM. *J. Vocat. Behav.* 98 152–162. 10.1016/j.jvb.2016.11.004

[B65] Ren S. Chadee D. (2017). Is guanxi always good for employee self-development in China? Examining non-linear and moderated relationships. *J. Vocat. Behav.* 98 108–117. 10.1016/j.jvb.2016.10.005

[B66] Ren S. Wood R. E. Zhu Y. (2015). *Business Leadership Development in China.* London: Routledge.

[B67] Robertson I. T. Jansen Birch A. Cooper C. L. (2012). Job and work attitudes, engagement and employee performance: where does psychological well-being fit in? *Leadersh. Organ. Dev. J.* 33 224–232. 10.1108/01437731211216443

[B68] Rossier J. Zecca G. Stauffer S. D. Maggiori C. Dauwalder J. P. (2012). Career adapt-abilities scale in a French-speaking Swiss sample: psychometric properties and relationships to personality and work engagement. *J. Vocat. Behav.* 80 734–743. 10.1016/j.jvb.2012.01.004

[B69] Rudisill J. R. Edwards J. M. Hershberger P. J. Jadwin J. E. McKee J. M. (2010). “Coping with job transitions over the work life,” in *Handbook of Stressful Transitions Across the Lifespan* ed. Miller T. W. (New York, NY: Springer), 111–131.

[B70] Rudolph C. W. Lavigne K. N. Zacher H. (2017). Career adaptability: a meta-analysis of relationships with measures of adaptivity, adapting responses, and adaptation results. *J. Vocat. Behav.* 98 17–34. 10.1016/j.jvb.2016.09.002

[B71] Russo M. Guo L. Baruch M. (2014). Work attitudes, career success and health: evidence from China. *J. Vocat. Behav.* 84 248–258. 10.1016/j.jvb.2014.01.009 24707977

[B72] Ryan R. M. Deci E. L. (2001). On happiness and human potentials: a review of research on hedonic and eudaimonic well-being. *Annu. Rev. Psychol.* 52 141–166. 10.1146/annurev.psych.52.1.141 11148302

[B73] Ryff C. D. Keyes C. L. M. (1995). The structure of psychological well-being revisited. *J. Pers. Soc. Psychol.* 69 719–727. 10.1037/0022-3514.69.4.719 7473027

[B74] Ryff C. D. Singer B. H. (2008). Know thyself and become what you are: a eudaimonic approach to psychological well-being. *J. Happiness Stud.* 9 13–39. 10.1007/s10902-006-9019-0

[B75] Salanova M. Del Líbano M. Llorens S. Schaufeli W. B. (2014). Engaged, workaholic, burned-out or just 9-to-5? toward a typology of employee well-being. *Stress Health* 30 11. 10.1002/smi.2499 23723156

[B76] Savickas M. L. (1997). Career adaptability: an integrative construct for life-span, life-space theory. *Career Dev. Q.* 45 247–259. 10.1002/j.2161-0045.1997.tb00469.x

[B77] Savickas M. L. (2002). *Career Construction: A Developmental Theory of Vocational Behavior. Career Choice and Development.* (San Francisco: Jossey-Bass), 149205

[B78] Savickas M. L. (2005). *The theory and practice of career construction. Career development and counseling: Putting theory and research to work*, Vol. 1 New York, NY: John Wiley, 42–70

[B79] Savickas M. L. (2013). *Career Construction Theory and Practice. Career Development and Counseling: Putting Theory and Research to Work*, Vol. 2 Hoboken, NJ: John Wiley and Sons, 144–180 10.4135/9781412952675

[B80] Savickas M. L. Nota L. Rossier J. Dauwalder J. P. Duarte M. E. Guichard J. (2009). Life designing: a paradigm for career construction in the 21st century. *J. Vocat. Behav.* 75 239–250. 10.1016/j.jvb.2009.04.004

[B81] Savickas M. L. Porfeli E. J. (2012). Career adapt-abilities scale: construction, reliability, and measurement equivalence across 13 countries. *J. Vocat. Behav.* 80 661–673. 10.1016/j.jvb.2012.01.011

[B82] Schaufeli W. B. Bakker A. B. Salanova M. (2006). The measurement of work engagement with a short questionnaire: a cross-national study. *Educ. Psychol. Meas.* 66 701–716. 10.1177/0013164405282471

[B83] Schaufeli W. B. Salanova M. González-Romá V. Bakker A. B. (2002). The measurement of engagement and burnout: a two sample confirmatory factor analytic approach. *J. Happiness Stud.* 3 71–92. 10.1023/A:1015630930326

[B84] Shimazu A. Schaufeli W. B. Kamiyama K. Kawakami N. (2015). Workaholism vs. work engagement: the two different predictors of future well-being and performance. *Int. J. Behav. Med.* 22 18–23. 10.1007/s12529-014-9410-x 24696043

[B85] Siu O. L. Spector P. E. Cooper C. L. Lu C. Q. (2005). Work stress, self-efficacy, Chinese work values, and work well-being in Hong Kong and Beijing. *Int. J. Stress Manag.* 12 274–288. 10.1037/1072-5245.12.3.274

[B86] Smith P. B. Huang H. J. Harb C. Torres C. (2012). How distinctive are indigenous ways of achieving influence? A comparative study of guanxi, wasta, jeitinho, and “pulling strings”. *J. Cross Cult. Psychol.* 43 135–150. 10.1177/0022022110381430

[B87] Sobel M. E. (1982). Asymptotic confidence intervals for indirect effects in structural equation models. *Sociol. Methodol.* 13 290–312. 10.2307/270723

[B88] Trefalt Š. (2013). Between you and me: setting work-nonwork boundaries in the context of workplace relationships. *Acad. Manag. J.* 56 1802–1829. 10.5465/amj.2011.0298

[B89] Tsui A. S. Farh J. L. (1997). Where guanxi matters: relational demography and guanxi in the chinese context. *Work Occup.* 24 56–79. 10.1177/0730888497024001005

[B90] Urbanaviciute I. Kairys A. Pociute B. Liniauskaite A. (2014). Career adaptability in Lithuania: a test of psychometric properties and a theoretical model. *J. Vocat. Behav.* 85 433–442. 10.1016/j.jvb.2014.09.005

[B91] Van Wingerden J. Bakker A. B. Derks D. (2017). Fostering employee well-being via a job crafting intervention. *J. Vocat. Behav.* 100 164–174. 10.1016/j.jvb.2017.03.008

[B92] Walsh W. B. Eggerth D. E. (2005). Vocational psychology and personality: the relationship of the five-factor model to job performance and job satisfaction. *Handb. Vocat. Psychol.* 3 267–295. 10.1016/j.pubrev.2006.08.002

[B93] Warner M. (2013). *Understanding Management in China: Past, Present and Future.* Milton Park: Routledge.

[B94] Wilson W. R. (1967). Correlates of avowed happiness. *Psychol. Bull.* 67:294 10.1037/h0024431 6042458

[B95] Wong Y.-T. Ngo H.-Y. Wong C. S. (2003). Antecedents and outcomes of employees’trust in Chinese joint ventures. *Asia Pac. J. Manag.* 20 481–499. 10.1023/A:1026391009543

[B96] Xie A. G. (2011). Investigation and thinking of happiness of college students. *Value Eng.* 5 255–257. 10.14018/j.cnki.cn13-1085/n.2011.15.144 22275281

[B97] Xin K. Pearce J. L. (1996). Guanxi: connections as substitutes for formal institutional support. *Acad. Manag. J.* 39 1641–1658. 10.2307/257072

[B98] Yang Q. (2013). Boosting happiness of the new generation of migrant workers in the new urbanization. *J. Guangzhou Univ.* 12 19–23.

[B99] Zacher H. (2014). Career adaptability predicts subjective career success above and beyond personality traits and core self-evaluations. *J. Vocat. Behav.* 84 21–30. 10.1016/j.jvb.2013.10.002

[B100] Zhang X. A. Li N. Harris T. B. (2015). Putting non-work ties to work: the case of guanxi in supervisor–subordinate relationships. *Leadersh. Q.* 26 37–54. 10.1016/j.leaqua.2014.04.008

[B101] Zheng X. Zhu W. Zhao H. Zhang C. (2015). Employee well-being in organizations: theoretical model, scale development, and cross-cultural validation. *J. Organ. Behav.* 36 621–644.

